# Selectively Imaging Cranial Sensory Ganglion Neurons Using AAV-PHP.S

**DOI:** 10.1523/ENEURO.0373-21.2022

**Published:** 2022-06-03

**Authors:** Andoni I. Asencor, Gennady Dvoryanchikov, Vivien Makhoul, Pantelis Tsoulfas, Nirupa Chaudhari

**Affiliations:** 1 Department of Physiology and Biophysics, University of Miami Miller School of Medicine, Miami, FL 33136; 2 Miami Project to Cure Paralysis, University of Miami Miller School of Medicine, Miami, FL 33136; 3 Department of Otolaryngology, University of Miami Miller School of Medicine, Miami, FL 33136

**Keywords:** AAV-PHP.S, auricular neurons, calcium imaging, labeling afferent fibers, pseudounipolar sensory neurons, somatosensory

## Abstract

Because of their ease of use, adeno-associated viruses (AAVs) are indispensable tools for much of neuroscience. Yet AAVs have been used relatively little to study the identities and connectivity of peripheral sensory neurons, principally because methods to selectively target peripheral neurons have been limited. The introduction of the AAV-PHP.S capsid with enhanced tropism for peripheral neurons (Chan et al., 2017) offered a solution, which we further elaborate here. Using AAV-PHP.S with GFP or mScarlet fluorescent proteins, we show that the mouse sensory ganglia for cranial nerves V, VII, IX, and X are targeted. Pseudounipolar neurons of both somatic and visceral origin, but not satellite glia, express the reporters. One week after virus injection, ≈66% of geniculate ganglion neurons were transduced. Fluorescent reporters were transported along the central and peripheral axons of these sensory neurons, permitting visualization of terminals at high resolution, and in intact, cleared brain using light sheet microscopy. Further, using a Cre-dependent reporter, we demonstrate by anatomic and functional criteria, that expression is in a cell type-selective manner. Finally, we integrate earlier neuroanatomical and molecular data with *in vivo* Ca^2+^ imaging to demonstrate the sensory characteristics of geniculate ganglion auricular neurons, which were previously undocumented. Our analyses suggest that the AAV-PHP.S serotype will be a powerful tool for anatomically and functionally mapping the receptive fields and circuits of the expanding numbers of molecular subtypes of many somatosensory and viscerosensory neurons that continue to be defined via single-cell RNA sequencing.

## Significance Statement

Adeno-associated virus (AAV) vectors are an essential tool for visualizing, manipulating, and recording the activity of neurons of the central nervous system. However, the technology is not widely used for peripheral neurons because of technical limitations. A recently introduced new serotype, AAV-PHP.S, targets peripheral neurons (Chan et al., 2017). Here, we establish key parameters for using this virus for the peripheral nervous system, including which cells are transduced, the timing of reporter expression in somata and transport into terminals ≥1 cm away. We demonstrate the accuracy of Cre-dependent constructs for cell type-selective expression and use this tool to record from a class of somatosensory ganglion neurons whose sensory characteristics have not previously been analyzed.

## Introduction

Adeno-associated viruses (AAVs) have emerged as one of the preferred tools of neuroscience research. Through their ease of use and effective delivery of cDNAs for fluorescent reporters and other proteins, AAVs have dramatically facilitated the mapping and manipulation of neural circuits in the brain (Samaranch et al., 2012; Nectow and Nestler, 2020). Depending on the serotype, AAVs are effective in different brain regions, selective for particular neuronal and/or glial sub-populations, and are transported in anterograde or retrograde direction in CNS neurons (Cearley et al., 2008; Ortinski et al., 2010; Salegio et al., 2013; Tervo et al., 2016). Stereotaxic injections at target sites combined with the use of virally delivered or transgenically expressed Cre- and Flp-recombinases allows constructing detailed maps of the projections of selected neuronal populations and their functional interactions (Rothermel et al., 2013; Saunders and Sabatini, 2015; Weinholtz and Castle, 2021).

To date, AAV-dependent manipulation of the peripheral nervous system has been much more limited, although many open questions about connectivity would benefit from this approach. One difficulty is that the somata of such neurons are contained in relatively inaccessible dorsal root, cranial or autonomic ganglia. The peripheral terminals of sensory neurons are distributed in skin, muscle or viscera, making it difficult to target a defined functional class of neurons. AAV-mediated transduction of peripheral neurons has been reported in a number of studies using various delivery methods such as injection into the sciatic nerve trunk (Towne et al., 2009), direct intraganglionic injection (Kollarik et al., 2010), and intrathecal infusion (Vulchanova et al., 2010; Schuster et al., 2013). However, these approaches are invasive, and the viral particles often lack wide tropism across peripheral neurons. Another approach is to inject virus near the peripheral terminals such as in and under epithelia, although this is inefficient if the goal is to transduce large numbers or many subtypes of peripheral neurons (Bloom et al., 2019; Taruno and Kashio, 2019).

A major advance came with the development of the synthetic neurotropic serotype, AAV.PHP.S, which was produced by directed evolution. Variations in the Cap gene of AAV9 were introduced, followed by iterative phenotypic selection for transduction of dorsal root ganglion (DRG) sensory neurons (Chan et al., 2017). The resulting AAV-PHP.S particles, when introduced into the circulation, were reported to infect mostly or only peripherally located neurons. Although Chan and colleagues reported that AAV-PHP.S displays tropism toward DRG and enteric neurons, they did not elaborate on other cranial ganglia with their distinct sensory neuron types. Nor were the central or peripheral projections of these peripheral sensory neurons examined in the original report. Here, we have systematically explored and report on transduction of neurons in the trigeminal, geniculate, petrosal and nodose ganglia (cranial nerves V, VII, IX, and X) using AAV-PHP.S. We also report on the time course for the expression of fluorescent reporters in neuronal somata and the axonal transport of fluorescence to peripheral and central terminals, which are at considerable distance. Further, we demonstrate using GCaMP and both anatomic and functional validation, that PHP.S viral particles can deliver reporters for stringent Cre-dependent expression, permitting exhaustive, or sparse labeling according to experimental needs.

## Materials and Methods

### Animals and tissues

All experiments were conducted according to the National Institutes of Health *Guidelines for the Care and Use of Laboratory Animals*, and protocols were approved by the University of Miami Institutional Animal Care and Use Committee. Mice of the following strains (Jax stock #) were purchased from The Jackson Laboratory and bred in-house: C57BL/6J (#000664), *Mafb*-2A-mCherry-2A-Cre (#029664), and *Penk*-IRES2-Cre (#025112). The *Mafb*-2A-mCherry-2A-Cre mice express both Cre and mCherry in many circulating and tissue-resident immune cells (X. Wu et al., 2016) as well as in a subset of neurons in the geniculate ganglion (Dvoryanchikov et al., 2017). *Penk*-IRES2-Cre mice express Cre in enkephalinergic neurons of the brain and spinal cord (François et al., 2017; Daigle et al., 2018) and also in the T3 subset of gustatory neurons of the geniculate ganglion (Dvoryanchikov et al., 2017). Mice of the *Plcb2*-GFP strain were produced and bred in-house and express GFP in the Type II cells found within all taste buds (Kim et al., 2006).

### Plasmids and AAV

Plasmids for producing AAV particles for GFP or GCaMP expression were obtained from Addgene, including pAAV-CAG-GFP (#37825), a gift from Edward Boyden, and pAAV.CAG.Flex.GCaMP6s.WPRE.SV40 (#100842), a gift from Douglas Kim. The cDNA for the red fluorescent protein, mScarlet-I (NCBI #KY021424; Bindels et al., 2017), was optimized based on human codon usage, then synthesized by GeneArt (ThermoFisher). This cDNA was used to replace the GFP sequence in pAAV-CAG-GFP (above) at BamHI and EcoRV sites.

Viruses were produced at the University of Miami viral core facility at the Miami Project to Cure Paralysis, using pUCmini-iCAP-PHP.S (Addgene #103006) in HEK293T cells. Titers of AAV-PHP.S preparations [in viral genomes (vg)/ml, assessed by qPCR] were as follows: CAG-GFP, 1.3 × 10^14^; CAG-mScarlet-I, 3.8 × 10^14^; and CAG-Flex-GCaMP6s, 2.9 × 10^14^.

Male or female mice between two and six months of age were retro-orbitally injected with 100-μl saline containing 1.3–3.8 × 10^12^ vg/mouse, using Terumo 1-ml tuberculin syringes with 26G × 3/8” needle. For the time course series, mice of both sexes were randomly assigned. For all experiments, virus was injected into the retro-orbital sinus. This route of injection offers the advantages of simple execution, minimal invasiveness, and rapid access to all peripheral tissues via the general circulation (Di Meo et al., 2017).

### Immunohistochemistry and imaging

For perfusion-fixation, mice were deeply anesthetized with ketamine and transcardially perfused sequentially with cold saline (0.9% NaCl), then 4% paraformaldehyde in saline. Tissues were dissected, postfixed for 1 h at 4°C (except overnight for brain and spinal cord), washed in PBS (3.8 mm NaH_2_PO_4_, 16.2 mm Na_2_HPO_4_, and 15 mm NaCl/1 l), cryoprotected overnight at 4°C in 30% sucrose in PBS, and embedded in OCT. Fixed cochleas, were decalcified for one week (Bas et al., 2019) before embedding in OCT.

Cryosections were cut on a Leica CM1900, sensory ganglia and lingual tissue to 20-μm thickness, spinal cord and brain to 40 μm. Sections, mounted on slides, were permeabilized (0.1% Triton X-100 in PBS), blocked (10% normal donkey serum) and incubated in diluted primary antibodies overnight at 4°C. After washing for 1 h in PBS, secondary antibody was incubated for 1–2 h. Sections were mounted under Fluoromount G (SouthernBiotech). Antibodies used and their concentrations are in Table 1.

**Table 1:** Primary and secondary antibodies used

Antigen	Antibody source, catalog	Lot #	Host	Dilution	Validation
**Primary antibodies**						
Cytokeratin 8	TROMA-I, DSHB, University of Iowa		Rat	1:1000	Loss of staining in knock-out tissue (Tao et al., 2003)
GFAP	ABClonal A0237	3515291101	Rabbit	1:100	Correct protein identified by immunoblot (Pan et al., 2021)
GFP	Invitrogen A11122	2015993	Rabbit	1:1000	No reaction in GFP-lacking tissue
mCherry	Abcam ab205402	GR3368071-6	Chicken	1:5000	No reaction in mCherry-lacking tissue
NeuN-Cy3	Millipore MAB377C3	2943768	Mouse	1:500	Staining on only neuronal nuclei and soma in ganglia
NTPDase2	J. Sévigny, Université Laval, Quebec	#mN2-36I6TG	Rabbit	1:1000	Correct protein identified by immunoblot (Bartel et al., 2006); loss of staining in knock-out tissue (Vandenbeuch et al., 2013)
P2X3	Millipore Labs AB5895	3227938	Rabbit	1:500	Loss of staining in knock-out tissue (Finger et al., 2005)
Phox2b	Santa Cruz sc-13224	C1016	Goat	1:500	2 independent Abs stain same nuclei (Dvoryanchikov et al., 2017)
Prrxl1/Drg11	Deolinda Lima, University of Porto		Rabbit	1:1000	Loss of staining in knock-out tissue (Rebelo et al., 2007)
βIII-tubulin	Santa Cruz, sc-80005 AF647 A2119		Mouse	1:50	Label selective for nerve fibers.
RFP (sections)	Synaptic Systems 390 005	1–3	Guinea pig	1:500	No reaction in RFP-lacking tissue
RFP (whole brain)	Rockland 600-401-379		Rabbit	1:1000	No reaction in RFP-lacking tissue

**Secondary antibodies**					
Rabbit IgG	Invitrogen A21207	2066086	Donkey	1:1000	Alexa 594
Rat IgG	Jackson 712-605-153	144825	Donkey	1:500	Alexa 647
Rabbit IgG	Invitrogen A31573	1874788	Donkey	1:1000	Alexa 647
Rabbit IgG	Invitrogen A21206	2072687	Donkey	1:1000	Alexa 488
Chicken IgY	Millipore AP194C	2865350	Donkey	1:1000	Cy3
Guinea Pig IgG	Jackson 706-165-148		Donkey	1:1000	Cy3
Goat IgG	Invitrogen A21447	2045332	Donkey	1:1000	Alexa 647
Rabbit IgG	ThermoFisher A32740		Goat	1:300	Alexa FluorPlus 594 (for whole-brain imaging)

All secondary antibodies validated by confirming lack of staining in absence of appropriate primary antibody.

Imaging was on an Olympus Fv1000 BX61 upright or an Olympus Fv1000 IX81 inverted laser scanning confocal microscope. Multichannel images were captured and were adjusted, only for brightness, in Photoshop. No contrast enhancement was applied. All images are shown at, or smaller than captured size.

### Quantifying efficiency of viral transduction

For GFP expressed from the CAG promoter, 20-μm-thick cryosections of geniculate ganglia were evaluated by intrinsic fluorescence of GFP and immunofluorescence for NeuN. A stack of confocal images was captured for each tissue section. To ensure that each GFP+ neuron was counted only once, only alternating sections were used, typically three to five per ganglion. Confocal images were Z-projected over 20 μm so that the shape and size of neurons (16–22 μm in diameter) was visible. We scored as positive, all cells which included a NeuN-immunoreactive nucleus, and which visually displayed GFP fluorescence above the background on the adjacent facial motor nerve track, were scored as positive.

For Cre-dependent expression of GCaMP in geniculate ganglia of *Mafb*-mCherry-Cre mice, 20-μm-thick cryosections of geniculate ganglia were immunostained for GFP, mCherry, and Phox2b, a marker of gustatory neurons (Dvoryanchikov et al., 2017). GFP+ neurons were evaluated for whether GFP exactly overlapped mCherry or conversely, surrounded a Phox2b-immunoreactive nucleus.

To quantify the number of fungiform and circumvallate taste buds that contained GFP-labeled gustatory fibers, 20-μm-thick cryosections were imaged (one image per section) for intrinsic fluorescence of GFP, and immunofluorescence for P2X3 and Ker8. Ker8 served to define the boundary of each taste bud. A bud was scored as containing GFP+ fibers only if the GFP signal exactly overlapped with P2X3-immunoreactivity within its boundary.

To quantify the intensity of GFP in fibers within fungiform taste buds, we used the same images as those used for taste buds. A stack of confocal planes was viewed in ImageJ, and a region of interest (ROI), representing the taste bud, was drawn based on Ker8 immunoreactivity. The stack was flattened and average intensity of GFP fluorescence within the ROI, relative to the area of the taste bud, was calculated. Because circumvallate taste buds are tightly packed in the epithelium, we could not accurately define the boundaries of individual buds in the z-dimension. Hence, this analysis of fluorescence intensity was performed only on fungiform, not on circumvallate taste buds.

### *In vivo* Ca^2+^ imaging

Mice were anesthetized with ketamine and xylazine, the geniculate ganglion was exposed via a dorsal approach and imaged for GCaMP6s as previously described (A. Wu et al., 2015; Leijon et al., 2019). In *Mafb*-mCherry-Cre mice, where Cre is expressed in auricular neurons, the following mechanical stimuli were applied to the dorsal aspect of the rigid base of the pinna, each for 5 s: a puff of compressed air, stroking with a bristle brush, gentle touch with a flat metal spatula, deflection with the same spatula, deep pressure with flat tweezers. In some instances, we also employed an upward flick of the pinna or brushing the whiskers, the latter as a negative control. Taste stimuli were perfused through the oral cavity as described (A. Wu et al., 2015; Leijon et al., 2019), and included 300 mm sucrose (sweet), 250 mm NaCl (salty), 10 mm citric acid (sour), a mix of 1 μm cycloheximide + 0.3 mm quinine (bitter), 20 mm monosodium glutamate + 1 mm inosine monophosphate (umami) in sequence. In *Penk*-Cre mice, the following acid stimuli were perfused through the oral cavity: 10 mm citric acid, 10 mm HCl, and 30 mm acetic acid. Some of the same mechanical stimuli as for mechanosensory neurons were used as negative controls.

For all stimulations, time series of fluorescence images were analyzed using ImageJ as previously described (A. Wu et al., 2015; Leijon et al., 2019) and are presented as changes of fluorescence normalized to baseline (i.e., ΔF/F_0_), for individual ROIs, each representing a single neuron. The F_0_ (i.e., baseline) value is calculated as mean fluorescence across 10–20 data points before stimulus onset. Increased fluorescence was scored as a neural response only if ΔF/F_0_ was above 3× SD of baseline and sustained for the duration of the stimulus.

### Tissue clearing and light sheet microscopy

For whole brain staining and clearing, we used an enhanced version of iDISCO (Renier et al., 2014; Bray et al., 2017; http://lab.rockefeller.edu/tessier-lavigne/assets/file/whole-mount-staining-bench-protocol-january-2015.pdf). Dissected whole brain was dehydrated through a methanol/PBS series, bleached overnight at 4°C, rehydrated, permeabilized at 37°C for 2 d, blocked for 2 d and then incubated with anti-RFP at 37°C for 10 d. After washing overnight, the brain was incubated in secondary antibody for 10 d, washed, dehydrated and cleared as described previously.

After clearing, samples were imaged the same day using light-sheet microscopy (Ultramicroscope, LaVision BioTec) using a fluorescence macro zoom Olympus MVX10 microscope with a 2× Plan Apochromatic zoom objective (NA 0.50). Image analysis and 3D reconstructions were performed using Imaris v9.5 software (Bitplane, Oxford Instruments) after removing autofluorescence using the Imaris Background Subtraction function with the default filter width.

## Results

### Targeting sensory ganglion neurons

Gene delivery to DRG neurons was previously demonstrated using AAV-PHP.S. To explore whether this virus effectively targets both somatic and visceral sensory ganglion neurons, we injected ≈10^12^ vg of AAV.PHP.S::CAG-GFP into the retroorbital sinus of three wild-type mice and after one week, examined the trigeminal (cranial V), geniculate (cranial VII), petrosal (cranial IX), nodose (cranial X), and several DRGs. Of these, trigeminal and DRGs confer general somatic sensation; the petrosal and nodose are visceral sensory ganglia that innervate the posterior tongue, pharynx, and multiple viscera; the geniculate ganglion is hybrid, including both general somatic sensitivity for the pinna and a visceral contingent of neurons innervating fungiform taste buds of the anterior tongue (D’Autreaux et al., 2011; Dvoryanchikov et al., 2017).

**Figure 1.**
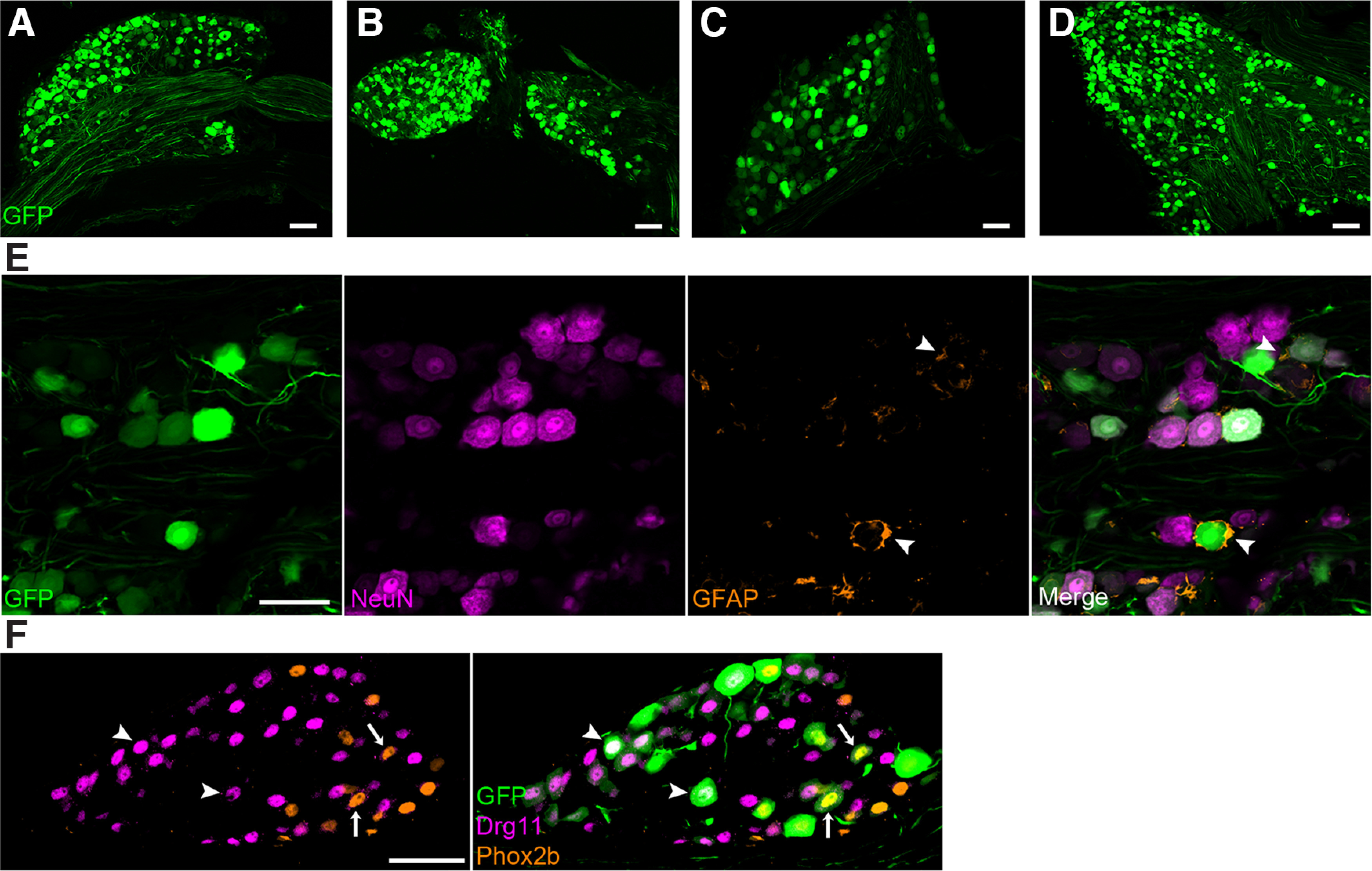
AAV-PHP.S transduces neurons in multiple sensory ganglia. AAV-PHP.S::CAG-GFP, injected into the retroorbital sinus of three mice, resulted in expression of GFP in (***A***) dorsal root, (***B***) nodose-petrosal complex, (***C***) geniculate, and (***D***) trigeminal ganglia, viewed for GFP intrinsic fluorescence in cryosections. Ganglia were dissected 7 d postinjection. ***E***, Cryosections of trigeminal ganglion (as in ***D***) were immunostained for NeuN (magenta) and GFAP (orange) to identify neurons and satellite glia, respectively. Only neurons are seen to express GFP. ***F***, Cryosections of geniculate ganglion (as in ***C***) were immunostained for Phox2b (orange) and Drg11 (magenta) to identify visceral and somatic neuronal nuclei, respectively. Several neurons of each class (arrowheads, somatic; arrows, visceral) are seen to express GFP (green). All images are single confocal plane. Scale bars: 50 μm. A small number of GFP+ cells were also observed in cochlea (Extended Data Fig. 1-1).

In cryosections of all ganglia, 7 d after injection and later, we observed expression of GFP in many neurons (Fig. 1*A–D*). The brightness of GFP fluorescence across neurons within a single ganglion was variable, which may reflect different numbers of viral particles infecting individual cells, or that some neuron types are more efficiently targeted. GFP expression was detected in each of the examined ganglia from three mice.

10.1523/ENEURO.0373-21.2022.f1-1 Extended Data Figure 1-1 AAV-PHP.S transduces a small number of cells in the spiral ganglion; 15-μm-thick cryosections of decalcified cochlea from mice infected with AAV-PHP.S::CAG-GFP were immunostained for βIII-tubulin (magenta). Those neurons which are robustly GFP-positive are also negative for βIII-tubulin (arrows). This suggests that AAV-PHP.S may be targeting the very few Type II pseudounipolar neurons present in the spiral ganglion, and avoiding the majority of βIII-tubulin-positive Type I bipolar neurons. Images are single plane. Scale bar: 20 μm Download Figure 1-1, TIF file.

To evaluate whether AAV.PHP.S was targeting glia in addition to neurons, we immunostained sections of trigeminal ganglion for NeuN, to detect neurons, and GFAP, which is expressed at low levels in a subset of the satellite glia which encase the ganglion neurons (Chudler et al., 1997; Villa et al., 2010). The distinctive crescent shape of satellite glia was not observed among GFP+ cells. GFP-expressing cells were consistently NeuN+, large and polygonal, and GFAP-negative (Fig. 1*E*). We also confirmed that GFP-expressing cells in the geniculate, petrosal, nodose and DRGs were shaped and sized like neurons and were NeuN+ (data not shown). Thus, AAV-PHP.S appears to target neurons of several distinct sensory ganglia, but not their satellite cells.

To assess whether both somatic and visceral sensory neurons were transduced, we immunostained geniculate ganglion cryosections for Phox2b and Drg11/Prrxl1, which are markers, respectively, for the viscerosensory gustatory neurons and the somatosensory auricular neurons in this ganglion (D’Autreaux et al., 2011; Dvoryanchikov et al., 2017). Across geniculate ganglia from four mice, both visceral and somatic neurons were GFP+ (Fig. 1*F*, arrows and arrowheads, respectively).

We did not observe GFP-label in the olfactory epithelium (data not shown). In a limited number of cochleas (two mice), we observed a small number of brightly fluorescent cells. These were intermingled among Type I spiral ganglion neurons (SGNs), identified by their location and immunoreactivity for βIII-tubulin (Extended Data Fig. 1-1). The GFP+ cells resemble Type II SGNs in their small numbers, location, size and lack of βIII-tubulin staining (Vyas et al., 2019).

### Maximal expression in ganglia by 7 d.

**Figure 2.**
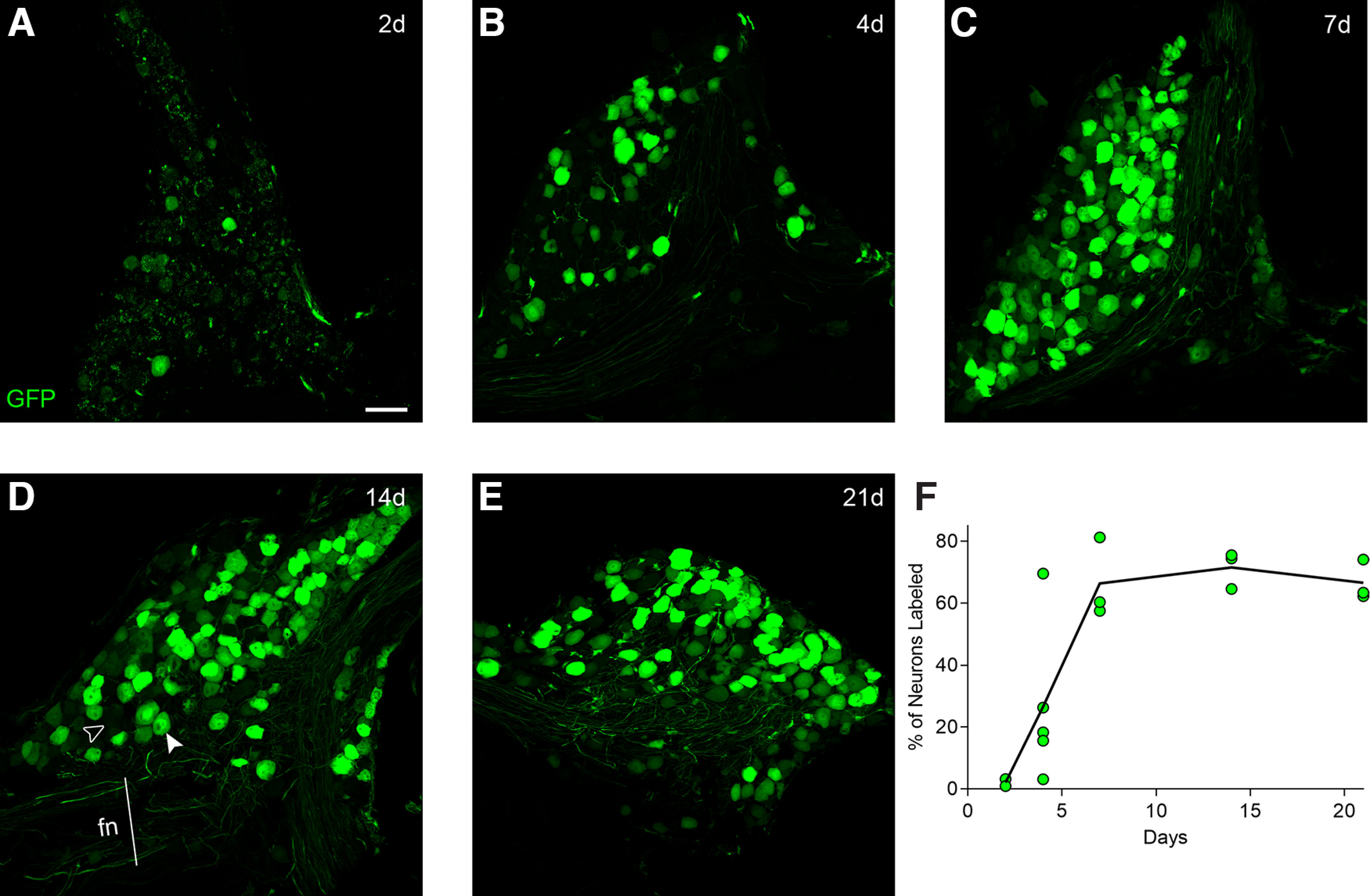
Time course of GFP expression. ***A–E***, Confocal images of cryosections of geniculate ganglia from mice injected with AAV-PHP.S::CAG-GFP, and analyzed at 2, 4, 7, 14, and 21 d postinjection. Intrinsic fluorescence of GFP was captured in parallel for all images; brightness was increased only in ***A*** to demonstrate very low-level expression at the earliest stage. ***F***, Transduction efficiency was quantified in confocal images of cryosections immunostained for NeuN. All neurons that displayed fluorescence visibly above background on the facial nerve (fn) track were scored as positive (solid arrowhead in ***D***). An example of a GFP-negative is indicated (open arrowhead in ***D***). Data are presented as the percentage of neurons that were GFP-labeled. Each symbol represents data from 400–600 neurons from a separate mouse (with 2, 5, 3, 3, 3 mice at successive time points). Solid line is the average for all mice at each time point. Scale bar: 50 μm.

To quantify GFP expression and its time course, we selected the geniculate ganglion. We retro-orbitally injected 16 wild-type mice with AAV-PHP.S::CAG-GFP (10^12^ vg/mouse), and examined them across a three-week time course (Fig. 2*A–E*). As early as 2 d postinjection, traces of GFP fluorescence could be detected in a few geniculate ganglion neurons. To quantify the efficiency of reporter delivery, we immunostained cryosections of individual geniculate ganglia for NeuN and scored the fraction of 400–600 NeuN+ cells per mouse that were GFP-labeled. By 7 d postinjection and subsequently, the fraction visible as GFP+ reached approximately two-thirds of all neurons in the ganglion, and showed a plateau (66 ± 7%, 72 ± 3%, 67 ± 4% at one, two, three weeks, respectively; mean ± SEM). This is comparable to the maximum frequency seen for most *in vivo* transduction by AAV (Aschauer et al., 2013). Several other strains of AAV also are reported to reach broad plateau levels of expression in most tissues 7–14 d after injection in the circulation (Zincarelli et al., 2008).

### Peripheral neuron selectivity

The use of AAV-PHP.S along with injection into the general circulation was reported to limit viral targeting to the peripheral nervous system (Chan et al., 2017). However, the report did not elaborate on how effective this restriction was. If AAV-PHP.S is to be useful for tracing the central projections of sensory afferent neurons, it is essential that there should be little to no labeling of resident neurons in areas where such central projections terminate. Thus, we examined the brainstem and spinal cord in areas that contain the central projections of sensory neurons targeted by AAV-PHP.S.

**Figure 3.**
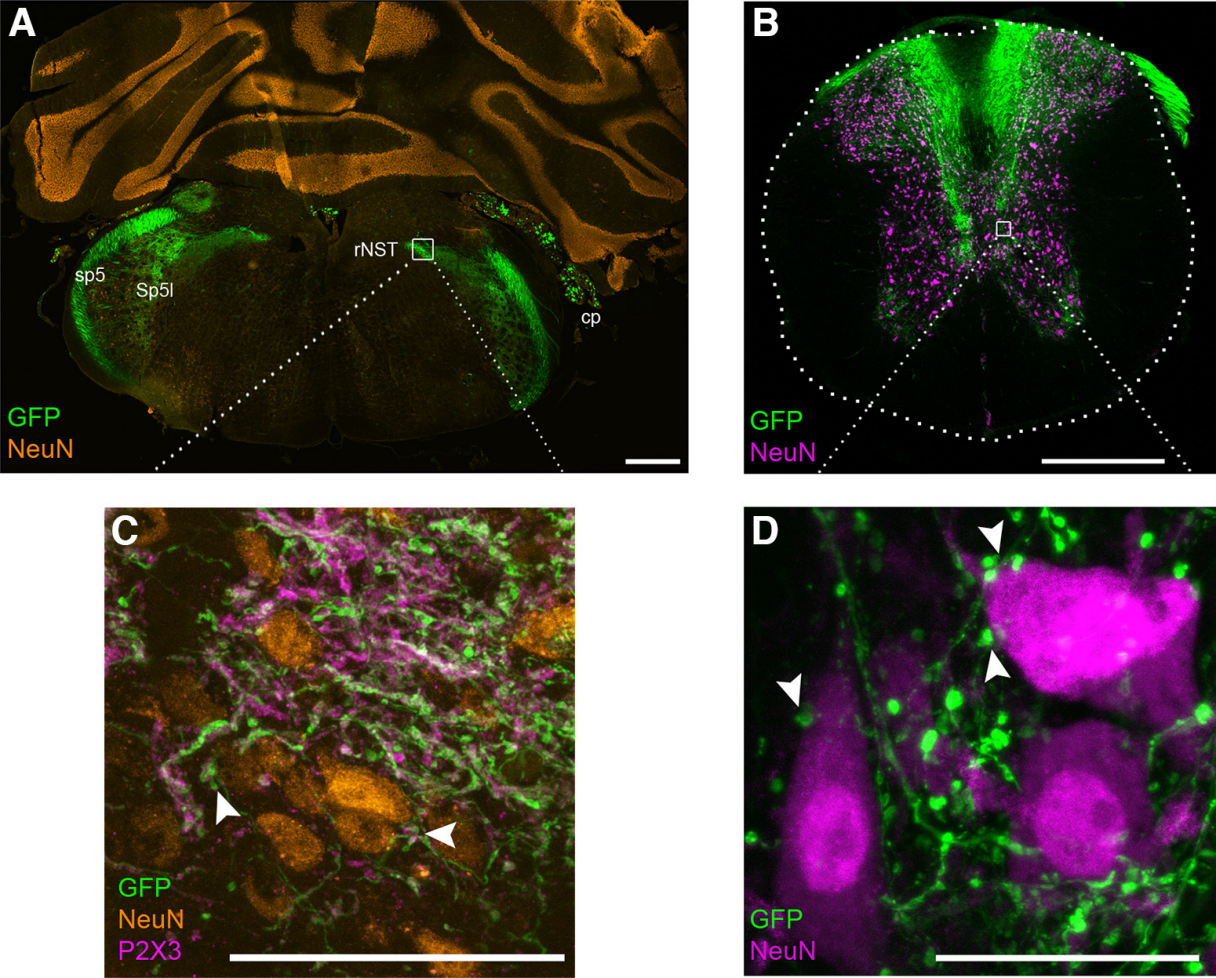
Only PNS neurons are targeted; CNS neurons remain unlabeled. Mice were injected with AAV-PHP.S::CAG-GFP and tissues were harvested 14 d later. ***A***, Cryosection of hindbrain, immunostained for NeuN (orange) shows that AAV-delivered GFP is absent from central neurons and limited to areas that receive afferent inputs from peripheral sensory neurons: the spinal trigeminal tract (sp5), spinal trigeminal nucleus interpolar (Sp5l) and rNST with its gustatory inputs. ***B***, Section of mid-thoracic spinal cord, imaged for NeuN immunoreactivity (magenta) and intrinsic fluorescence of AAV-delivered GFP, which is limited to sensory inputs from the DRG. ***C***, Higher magnification of boxed region of rNST, additionally stained for P2X3 (magenta) to label gustatory afferents. Many of the finest GFP+ fibers are gustatory, with boutons that suggest synapses on rNST neurons (arrowheads). ***D***, High magnification of a region of ventral horn indicated by a box in ***B***, where GFP+ afferent sensory fibers present boutons resembling synapses, juxtaposed against primary motor neurons (identified by their large size). Scale bars: 500 μm (***A***, ***B***) and 50 μm (***C***, ***D***).

In hindbrain sections from wild-type mice injected with AAV.PHP.S::CAG-GFP, GFP-labeled axons were clearly detected in the spinal trigeminal tract (Sp5) and in the nucleus of the solitary tract (NST; Fig. 3*A*). These areas contain, respectively, the central projections of sensory neurons from either the trigeminal ganglion or the geniculate, petrosal and nodose ganglia (Hamilton and Norgren, 1984). In the spinal cord also, GFP-labeled fibers were detected in the dorsal horn, with some GFP+ fibers extending ventrally past the central canal (Fig. 3*B*).

At higher magnification, both Sp5 and NST contained GFP+ fibers, many of which displayed boutons that resemble synapses (Fig. 3*C*). In the rostral NST (rNST), where gustatory afferents project, many GFP+ fibers were immunoreactive for P2X3, a known marker for gustatory neurons (Bo et al., 1999; Finger et al., 2005). In the spinal cord, GFP+ fibers from the DRGs terminated throughout the dorsal horn. A minority of GFP+ fibers continued ventrally and displayed boutons that were juxtaposed against large neuronal somata (Fig. 3*D*). The location and size of these suggest they are primary motor neurons with proprioceptor fibers terminating directly on them. Qualitatively similar labeling of central terminals was detected at 7 and 14 d postinjection.

Importantly, in the hindbrain and spinal cord (Fig. 3*C*,*D*), we found no GFP-labeled structures that were NeuN-immunoreactive or resembled neuronal somata in shape and size. The absence of GFP+ central neurons in all areas examined confirms the strictly peripheral targeting by AAV-PHP.S.

### Labeling peripheral afferent terminals

**Figure 4.**
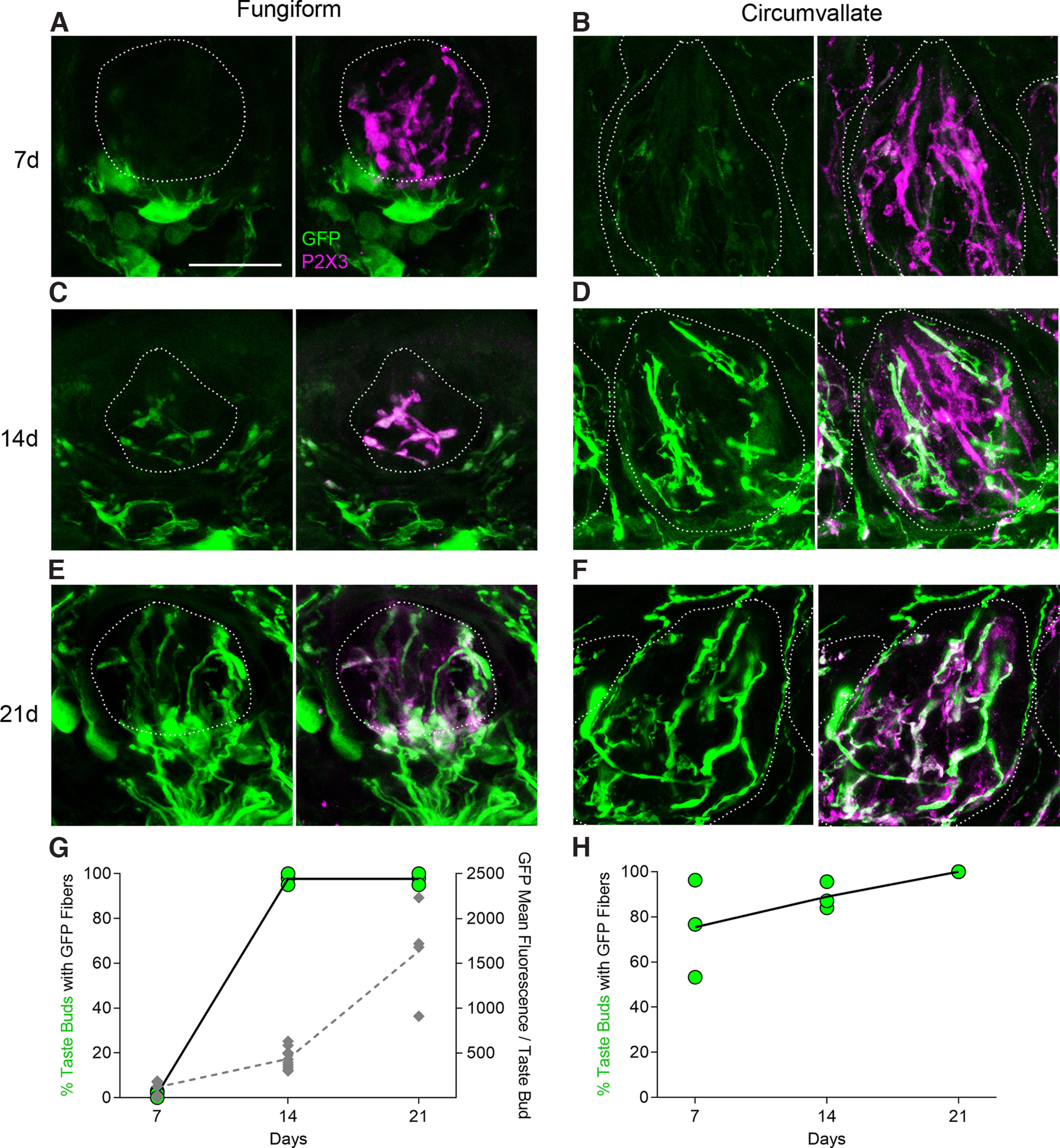
Peripheral terminals of sensory ganglion neurons are GFP-labeled. ***A–F***, Cryosections of fungiform (***A***, ***C***, ***E***) and circumvallate (***B***, ***D***, ***F***) taste buds from nine mice, injected with AAV-PHP.S;;CAG-GFP, were immunostained 7, 14, or 21 d postinjection to detect accumulation of axonally transported GFP to the peripheral terminals of sensory ganglion neurons. Dotted lines outline each taste buds (38–62 analyzed per mouse). Gustatory fibers are immunoreactive for P2X3 (magenta), allowing discrimination from P2X3-negative trigeminal fibers outside the taste buds. The number of GFP+ fibers visible and their fluorescence intensity appears to increase from 7 to 21 d. All images were captured in parallel at the same settings to make fluorescence intensities comparable. ***G***, ***H***, Quantification of incidence of GFP+ fibers within perimeter of taste buds at each time point. Each green symbol represents the fraction of 15–29 fungiform taste buds from one mouse (***G***) or 23–33 circumvallate taste buds from one mouse (***H***). Data from nine mice are included in each graph. In ***G***, the secondary *y*-axis depicts total fluorescence intensity of GFP+ fibers within each fungiform taste bud across the time course from 7 to 21 d. Each gray data point is a separate taste bud; a total of 20 fungiform taste buds from five mice were sampled for gray symbols. Scale bar: 20 μm.

Because high levels of GFP could be detected in ganglia by 7 d, we examined how rapidly fluorescent reporter could be detected in the sensory peripheral terminals. We selected for analysis, fungiform and circumvallate taste buds, which may be as much as 2 cm from the somata of the neurons that innervate them. Cryosections of lingual papillae from nine wild-type mice injected with AAV.PHP.S::CAG-GFP virus were immunostained for Ker8 and P2X3 to visualize taste buds and gustatory afferent fibers, respectively. At 7 d postinjection, some nerve fibers in circumvallate taste buds exhibited faint fluorescence, while fungiform taste buds showed no GFP+ fibers (Fig. 4*A*,*B*). Over the course of two additional weeks (Fig. 4*C–F*), fibers within taste buds became more numerous and acquired brighter fluorescence. We confirmed that all GFP-labeled structures within taste buds displayed co-expression with P2X3, and thus could be classified as gustatory fibers.

To quantify this increase of GFP+ fibers in taste buds, we employed two measures. First, we evaluated the fraction of taste buds that included GFP+ fibers. In the anterior tongue, GFP-labeled fibers were detected in very few fungiform taste buds at one week but were readily apparent in all buds by two weeks postinjection (Fig. 4*G*, green symbols). In the posterior tongue, labeled fibers were detected in most circumvallate taste buds one week after injection (Fig. 4*H*). This delay for the anterior tongue may represent the speed of slow axonal transport of GFP for the additional ≈6- to 8-mm transport needed to reach fungiform taste buds. A second measure employed was the mean intensity of GFP per fungiform taste bud. Gray symbols (Fig. 4*G*) illustrate the progressive accumulation of fluorescence in each bud, as GFP was transported to terminals.

In all lingual sections (Fig. 4*A*,*B*), we observed large GFP-expressing cells located in connective tissue below the taste bud and epithelium. Their location and morphology suggest resident macrophages or dendritic cells, although we did not characterize them further. These cells became GFP+ well before GFP is detected in fibers innervating the epithelium.

**Figure 5.**
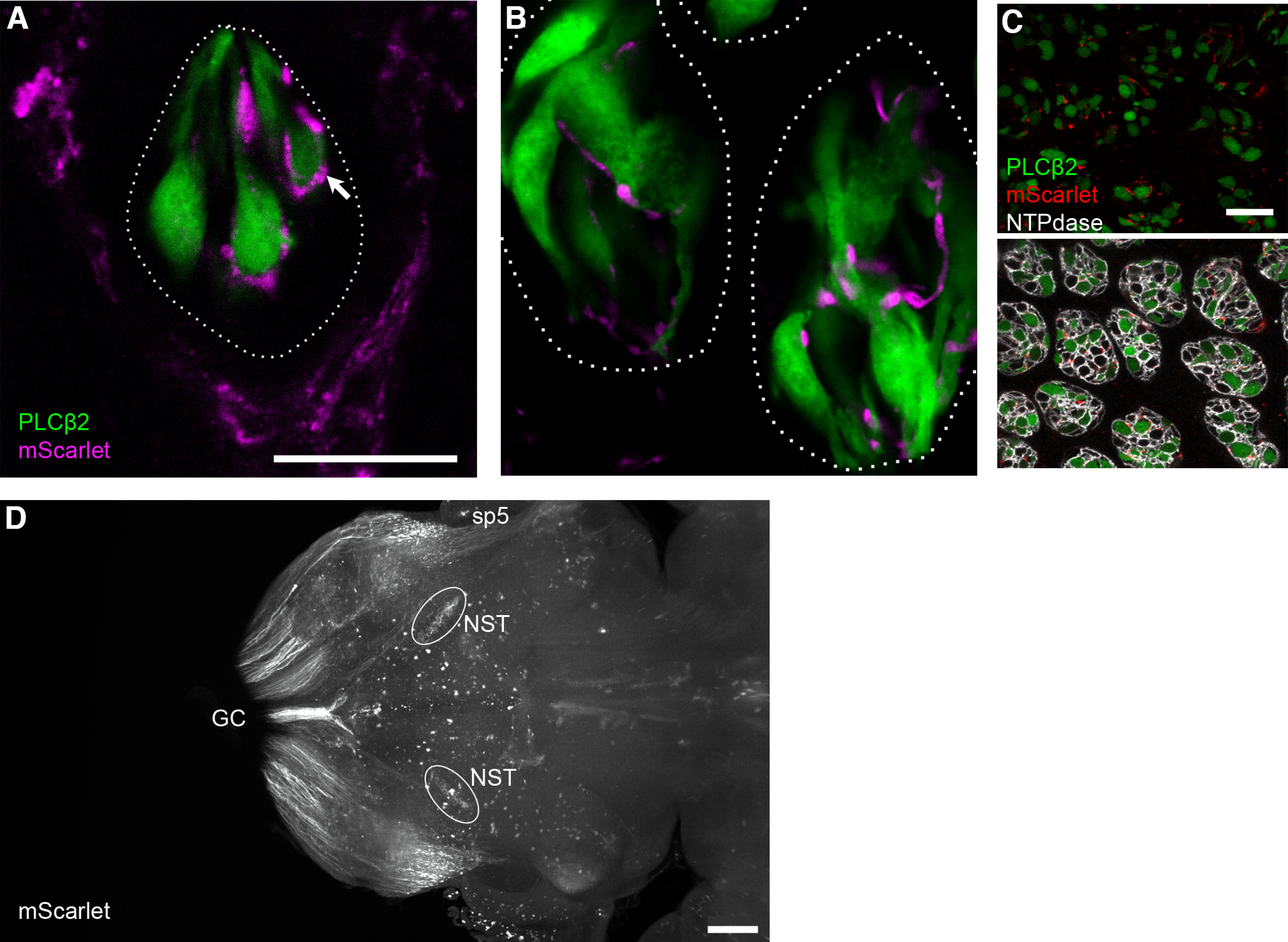
mScarlet reporter for visualizing individual fibers and fiber tracts. ***A–C***, Fungiform and circumvallate taste buds from a *Plcb2*-GFP mouse, injected with AAV-PHP.S::CAG-mScarlet-I, examined 21 d postinjection. In a single confocal plane of a fungiform papilla (***A***), two types of mScarlet+ nerves (magenta) are visible: trigeminal fibers that form a corona around the taste bud, and gustatory fibers that enter the taste bud. Terminal boutons of gustatory nerves are seen associated with individual Type II (GFP-expressing, green) chemoreceptor cells (arrow). ***B***, In a single confocal plane, two circumvallate taste buds similarly show gustatory afferents associating with GFP+ chemosensory cells. ***C***, Circumvallate taste buds from the same mouse as in ***A***, ***B***, viewed in cross-section and at low magnification, nearly every taste bud includes multiple afferent fibers that are mScarlet-labeled (red). Immunoreactivity for NTPDase2 outlines each taste bud cell. ***D***, Light-sheet microscope imaging of a cleared brain of a mouse injected with AAV-PHP.S;;CAG-mScarlet-I. Brain was viewed in a horizontal orientation. This image is a Z-projection through a 100-μm thickness that includes the rNST. The sensory afferent fibers of AAV-transduced neurons detected here include the gracile and cuneate sensory tracts (GC) entering the medulla caudally, the spinal trigeminal tract (sp5) and the terminals of gustatory afferents in the rNST. Scale bar: 20 μm (for taste buds) and 500 μm (for brain). A 3D reconstruction of the mScarlet-labeled hindbrain is in Movie 1, and permits visualization of the entry of multiple sensory afferents.

We also tested another fluorescent reporter, mScarlet-I, which is bright and highly suitable for imaging cleared whole brain, and offers a second color for dual-label transductions. We injected AAV.PHP.S::CAG-mScarlet-I into *Plcb2-*GFP mice in which the Type II chemosensory cells of all taste buds express GFP (Kim et al., 2006). Juxtapositions of individual Type II cells with individual afferent fibers could readily be visualized in fungiform (Fig. 5*A*) and circumvallate (Fig. 5*B*,*C*) taste buds. In both locations, mScarlet-labeled boutons on afferent fibers appear to terminate on Type II cells, and may represent afferent synapses (arrow). If AAV-delivered GFP or mScarlet-I were expressed selectively in a single neuron type from the ganglion, the method would permit precise definition of neuron-target interactions.

To visualize primary sensory fiber tracts in the brain in a 3D manner, we subjected intact brains from mice injected with AAV.PHP.S::CAG-mScarlet-I to immunostaining to enhance mScarlet fluorescence, followed by clearing and imaging by light sheet microscopy (Fig. 5*D*). Ascending spinal sensory tracts as well as trigeminal and gustatory tracts could readily be traced from their entrance into the CNS to their terminals in respective sensory nuclei (Fig. 5*D*; Movie 1).

Movie 1. 3D movie, sensory fiber tracts in hindbrain. 3D light sheet microscopy of hindbrain from mouse, 19 d after retroorbital sinus injection with AAV-PHP.S::CAG-mScarlet-I. Movie is a series of horizontal 2D images, representing a thickness of ≈1.5 mm. AAV-delivered mScarlet fluorescence is enhanced with anti-RFP. Ascending spinal sensory afferents of the Gracilis and Cuneate tracts enter from the right. Cranial afferents readily visible include the spinal trigeminal tract (sp5) and gustatory afferents terminating in the NST. 10.1523/ENEURO.0373-21.2022.video.1

### Cell type-selective expression for functional analysis

**Figure 6.**
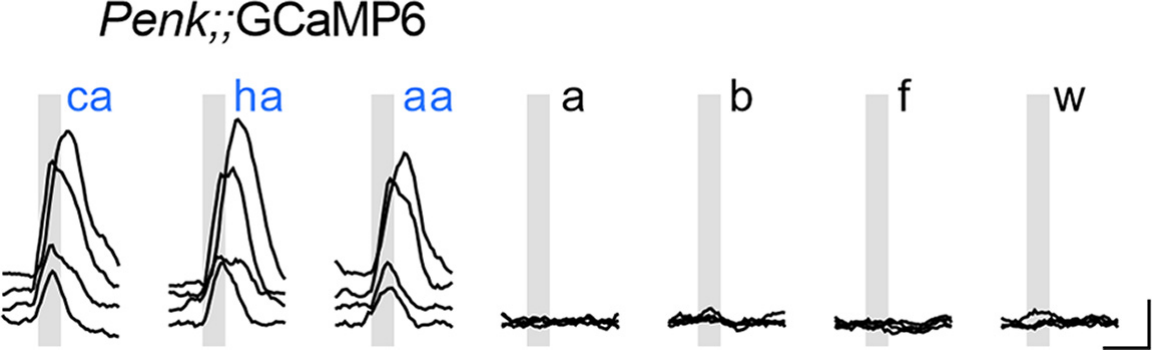
Ca^2+^ imaging of gustatory neurons with Cre-dependent GCaMP in a *Penk*-Cre mouse. Geniculate ganglion neurons were imaged under anesthesia 14 d after injection with AAV-PHP.S::*flex*-GCaMP6s. Traces are the responses (ΔF/F_0_) of four representative GCaMP+ neurons to oral perfusion of three acidic taste stimuli (gray bars): citric acid (ca), HCl (ha), acetic acid (aa), followed by mechano-stimulation of the whiskers (w) or pinna (as in Fig. 7) with a puff of compressed air (a), stroking with a bristle brush (b), and a flick (f). None of the mechanical stimuli elicited a response. Scale bar: 10 s, 1.0 ΔF/F_0_.

To confirm that AAV-PHP.S could be used to express sufficient concentration of Ca^2+^ reporter for functional imaging, we employed a Cre-dependent construct, AAV-PHP.S::CAG-flex-GCaMP6s and injected 3 × 10^12^ vg into the retroorbital sinus of *Penk*-Cre knock-in mice. In a previous study using transgenically expressed GCaMP, Penk-expressing neurons in the geniculate ganglion were reported to be selectively responsive to oral sour stimuli (Zheng et al., 2019). We exposed the geniculate ganglion and recorded neuronal responses by imaging GCaMP fluorescence. Because baseline fluorescence of GCaMP6 is very low, it was not possible to independently verify how many neurons were AAV-transduced. Nevertheless, 11 neurons (across two mice), responded to each of three acid stimuli applied orally (citric, hydrochloric, and acetic acid; Fig. 6). Repeated applications of these acid stimuli yielded repeatable responses. This first experiment served as a proof of concept for functional recordings, demonstrating (1) that GCaMP was expressed in a Cre-dependent fashion in Penk-Cre mice and (2) confirming the stimulus-specificity reported earlier. Because certain neurons in the geniculate ganglion are reported to be mechanosensitive, we also subjected these mice to several mechanical stimuli during the recording. None of the 11 acid-sensitive neurons responded to any mechanical stimuli, nor did any other neurons (of 36 total in the imaging fields) respond to mechanical stimuli.

### Using AAV-PHP.S to examine the sensory characteristics of auricular neurons

The “auricular” neurons of the geniculate ganglion were so named on the basis of clinical observations of herpes virus reactivation (Hunt, 1907), and neuroanatomical tracing (Semba et al., 1984). Yet, we are unaware of a functional demonstration of evoked responses by stimulation of the ear. Thus, we used AAV-PHP.S::CAG-flex-GCaMP6s to determine whether these neurons are functionally auricular and respond as expected for somatosensory neurons. Because about half the neurons in the geniculate ganglion were postulated to be auricular (D’Autreaux et al., 2011) and express Mafb (Dvoryanchikov et al., 2017), we injected the Cre-dependent AAV in *Mafb-*mCherry-Cre mice.

**Figure 7.**
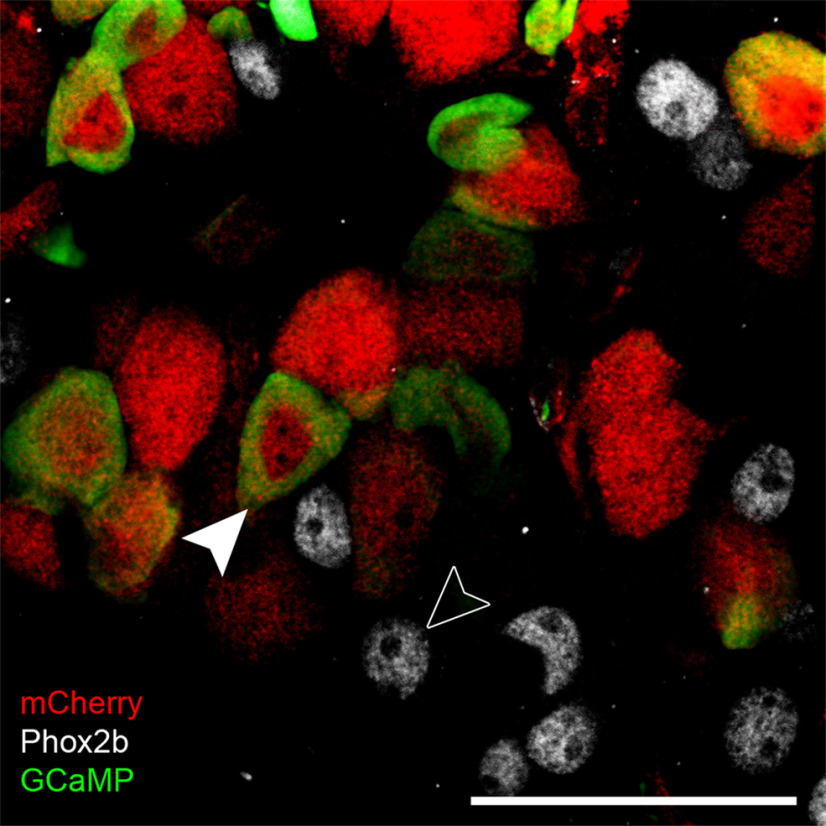
Cre-dependent expression in peripheral sensory neurons of a *Mafb-*mCherry-Cre mouse. The geniculate ganglion was examined 14 d after injecting AAV-PHP.S::*flex*-GCaMP6s. Cryosections were immunostained with anti-GFP (to detect GCaMP), anti-mCherry, and anti-Phox2b (to detect gustatory neurons). GCaMP6 (green) is detected only in neurons that express Cre and mCherry (red, filled arrowhead). The overwhelming majority of gustatory neurons in the ganglion lack mCherry and Cre, have Phox2b+ nuclei and did not express GCaMP (open arrowhead). Scale bar: 50 μm. The lack of GCaMP expression in central neurons that express Cre is shown in Extended Data Figure 7-1.

Before attempting functional imaging, we first validated the selective expression of GCaMP reporter in the Mafb-expressing subtypes of geniculate ganglion neurons, using molecular criteria. We injected 1.5–3.0 × 10^12^ vg of AAV-PHP.S::CAG-flex-GCaMP6s into each of four *Mafb-*mCherry-Cre knock-in mice. In the geniculate ganglion, Mafb is expressed in ≈half of all neurons. Of these, ≥90% are of the auricular class while ≤10% are large, oral-mechanosensory neurons (T2 class; Dvoryanchikov et al., 2017). We evaluated both the efficiency (fraction of Mafb+ neurons labeled) and accuracy (fraction of GCaMP-labeled neurons that lack Mafb) of the Cre-dependent AAV. For this, we immunostained cryosections of geniculate ganglia from *Mafb-*mCherry-Cre mice, 14 d after injection. 280–400 neurons were scored for each mouse and categorized as auricular (Phox2b-neg, mCherry+), T2 (Phox2b+, mCherry+) or gustatory (Phox2b+, mCherry-neg). GCaMP expression was almost completely limited to Cre-expressing neurons (Fig. 7, white arrowheads). Specifically, 442 of 929 mCherry+ neurons expressed GCaMP, whereas 429 of 431 Cre-lacking (i.e., gustatory) neurons lacked GCaMP+ expression (Fig. 7, open arrowhead). Thus, the Cre-dependent virus was ≈48% efficient in labeling Cre-expressing neurons and 99.5% accurate in sparing Cre-lacking neurons. The difference in incidence of GCaMP expression in Cre-expressing and Cre-lacking neurons is highly significant (*p* < 0.0001; Fisher’s exact test).

10.1523/ENEURO.0373-21.2022.f7-1 Extended Data Figure 7-1 Cre-dependent expression is limited to peripheral neurons. A *Mafb-*mCherry-Cre mouse was injected with AAV-PHP.S::*flex*-GCaMP6s and perfusion fixed 19 d later. ***A***, Cryosection of hindbrain shows mCherry+ (i.e., Cre-expressing) central neurons of the spinal trigeminal nucleus, oral part (Sp5O) and the ventral cochlear nucleus (VCN). These remain untransduced and completely devoid of GFP. Some GCaMP6 is visible in the choroid plexus (cp) within the lateral recess of the 4th ventricle. ***B***, Cryosection of thoracic spinal cord similarly shows many Cre-expressing neurons of the spinal gray and these remain untransduced. In both cases, the peripheral afferents lack Mafb and Cre expression, and thus also are unlabeled. Scale bars: 250 μm. Download Figure 7-1, TIF file.

Given a recent report of Cre-independent low-level expression in central neurons following AAV injection of a double-inverted GFP construct (Botterill et al., 2021), we further confirmed strict Cre-dependence of AAV-PHP.S::CAG-flex-GCaMP6s. No GCaMP expression was detected in the geniculate ganglion of a wild-type mouse that lacked Cre recombinase (data not shown).

Transduction with AAV-PHP.S::CAG-Flex-GCaMP6s was limited to peripheral neurons. Mafb-expressing neurons are present in the hindbrain, in the ventral cochlear nucleus and spinal trigeminal nucleus (Lein et al., 2007; http://mouse.brain-map.org/gene/show/16430) and scattered throughout the dorsal horn of the spinal cord. While expression of the mCherry transgene was evident, such central neurons remained unlabeled with GCaMP6 (Extended Data Fig. 7-1).

Following the molecular and morphologic validation of cell-type specificity, we imaged Ca^2+^ responses of geniculate ganglion neurons *in vivo* as previously described (A. Wu et al., 2015; Leijon et al., 2019).

**Figure 8.**
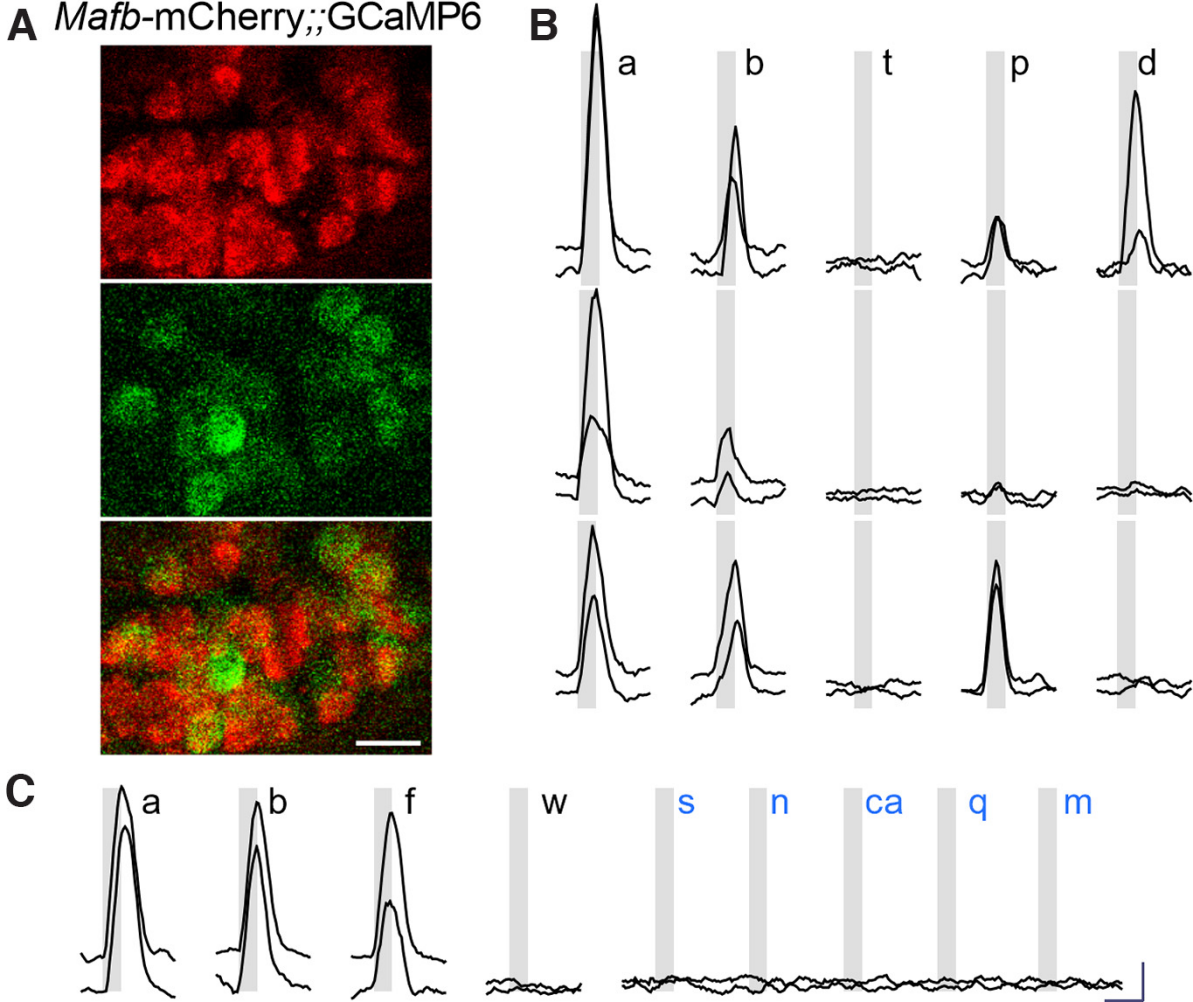
Ca^2+^ imaging of auricular neurons with Cre-dependent GCaMP in *Mafb-*mCherry-Cre mouse. ***A***, Geniculate ganglion of anesthetized mouse, 14 d after injection with AAV-PHP.S::*flex*-GCaMP6s, viewed for mCherry (red) and GCaMP (green) during recording. This region of the ganglion includes a high density of auricular (mCherry+) neurons, many of which express GCaMP. ***B***, Responses (ΔF/F_0_) of 6 GCaMP+ auricular neurons from a different ganglion, similar to ***A*,** to five types of mechano-stimulation of the rigid, cartilaginous portion of the pinna. Stimuli (gray bars) included from left to right, a puff of compressed air (a), stroking with a bristle brush (b), gentle touch with flat spatula (t), deep pressure with same spatula (p), and deflection of pinna with same spatula (d). Example traces of two neurons that responded in each of three different patterns are shown. ***C***, Responses of two GCaMP+ auricular neurons, stimulated first with mechanical stimuli as in ***B***, then by flicking the pinna (f) or whiskers (w), and finally with oral perfusion of five stereotypical taste stimuli in the mouth. Zero of 49 neurons stimulated in this manner (across 4 mice) responded to whisker stimulation or oral tastants: sucrose (s), NaCl (n), citric acid (ca), quinine/cycloheximide (q) and MSG/IMP (m). Scale bars for ***B***, ***C***: 10 s, 1.0 ΔF/F_0_.

We stimulated the pinna with 6 different mechanical stimuli, including a puff of compressed air as a search stimulus followed by stroking with a stiff bristle brush, gentle touch, deep pressure, deflection and flicking. Neurons that were mCherry+ and GCaMP+ (Fig. 8*A*; i.e., mostly auricular) responded robustly and repeatedly to brushing, and flicking, but not to gentle touch (Fig. 8*B*; 49 neurons from three mice). Responses to deeper pressure and deflection of the pinna were not consistent even for a given neuron (Fig. 8*B*). None of these neurons responded to warm (45°C) and cold (9°C) temperatures (data not shown). In addition, auricular neurons did not respond to the stimulation of whiskers, which represent a different receptive field (Fig. 8*C*). Importantly, all 49 neurons were refractory to taste stimuli, applied orally. In the same imaging fields, we detected no responses to either mechanical or taste stimuli, supporting our observation that only auricular (i.e., Mafb and Cre-expressing) neurons were GCaMP+. These experiments constitute the first functional characterization of auricular neurons to somatosensory stimulation and demonstrate that the geniculate ganglion includes a restricted set of somatosensory neuron types.

## Discussion

The creation of AAV-PHP.S (Chan et al., 2017) introduced the possibility of transducing peripheral neurons with a variety of reporters and/or gene products for anatomic and physiological studies. We have elaborated on the original study by demonstrating that in addition to DRG and enteric neurons, the neurons of many cranial ganglia, including both visceral and somatic classes also can be transduced. We show that virally delivered GFP is visible in neuronal somata within 2 d, reaching a maximum number of neurons and brightness within a week. We did not observe GFP expression in satellite cells in ganglia or Schwann cells along nerve trunks. Soluble fluorescent proteins, GFP, mScarlet, mCherry, were transported along the axons of sensory neurons at similar rates. Among the peripheral sensory neurons we examined, all those transduced by AAV-PHP.S (DRG, trigeminal, geniculate, petrosal, vagal) were pseudo-unipolar. The majority of SGNs (which are bipolar) showed no evidence of GFP. A scattered few neurons in the cochlea were GFP+, and we infer these might be Type II cochlear afferents, which are pseudounipolar. Importantly, we demonstrate that PHP.S viral particles can be used for Cre-dependent expression for accurate cell type-selective targeting. We employed this last feature to document the functional responses of a class of neurons termed “auricular,” and to show that these neurons represent a restricted set of somatosensory neuron types, innervating the pinna of the ear.

Over several decades, enzymes, dextrans, and viruses have been developed for neuronal tracing of central circuits, based on transport in anterograde (from somatodendritic compartment down the axon) or retrograde (from axon terminal to soma). For AAV, particular natural and engineered serotypes exhibit transport in one or the other, or both directions (McFarland et al., 2009; Aschauer et al., 2013; Rothermel et al., 2013; Castle et al., 2014; Tervo et al., 2016). The directionality of AAV transport relies on both viral serotype and microtubule-based mechanisms and adapters in dendrites and axons (Leopold and Pfister, 2006). However, pseudounipolar sensory neurons such as those found in dorsal root, trigeminal, gustatory, and other visceral ganglia present a rather different version of polarity than that defined for central neurons (Nascimento et al., 2018; Shorey et al., 2021). These neurons have no dendrites, only a single axon that exits the soma, bifurcating into one axonal branch toward the peripheral receptive field and the other toward central target(s). Because we introduced AAV-PHP.S into the bloodstream and cranial ganglia exist outside the blood-brain barrier, it seems likely that the virus gains entry at or near the sensory neuronal soma. The rapid (within 2 d) expression of fluorescent reporters in somata (Fig. 2) is consistent with this. The GFP reporter was detected along axons with progressively longer delay as distance from the soma increases (Fig. 4). We calculate a rate typical of slow axonal transport (≈0.5–1.5 mm/d), based on the distance of circumvallate or fungiform taste buds from the geniculate ganglion (≈5 and 15 mm, respectively). Transport along both axonal directions is at roughly similar velocity.

Although AAV9 (the parental virus for AAV.PHP.S) transduces astrocytes in adult brain (Gombash et al., 2014), we found that AAV-PHP.S did not transduce GFAP-expressing satellite glia in ganglia. We did, however, observe some cells immediately below lingual epithelium that were rapidly transduced (within 2 d of virus injection) that might be either immune or Schwann cells (Fig. 4*A*).

While many detailed connectivity maps of central pathways have been built using AAV, these tools have been applied much less to peripheral sensory neurons. Instead, neuroanatomically mapping the spinal and brainstem targets of sensory ganglion neurons has relied on tracers and well-defined molecular markers of neuronal types. This is the case even for recently identified sensory neuron subtypes (Häring et al., 2018; Oliver et al., 2021; H. Wu et al., 2021). The use of AAV-PHP.S, particularly in combination with Cre-dependent expression, enhances the experimenter’s toolbox, allowing precise and efficient labeling of peripheral neurons while leaving resident neurons in the central target field unlabeled. With the recent molecular definition of molecular subtypes of peripheral sensory neurons (Dvoryanchikov et al., 2017; Vyas et al., 2019; Zheng et al., 2019; Handler and Ginty, 2021; Xing et al., 2021), we anticipate this method will find substantial utility. While Cre-dependent transgenic expression of reporters has been used for many of these, AAV offers advantages such as avoiding developmental mis-labeling of neurons and using sparse labeling for tracing individual neurons to their central or peripheral targets. The use of Cre-dependent AAV-PHP.S constructs with functional reporters should open new directions for neuroanatomically and functionally defining sensory submodalities in the somatosensory as well as gustatory systems. For the taste system in particular, there is a critical need to define the brainstem targets of molecular classes of gustatory neurons. Controversies regarding the neuroanatomical basis for taste coding from the periphery to first central relays will benefit from these novel mapping tools. The resolution afforded by visualizing soluble, and synaptically targeted fluorescent reporters in individual fibers making synapses on defined peripheral receptors and rNST neurons (similar to Fig. 3*C*) will allow the development of accurate connectivity maps.

Using AAV-PHP.S, we report the sensory characteristics of auricular neurons of the geniculate ganglion. These were originally designated “auricular” based on clinical cases of herpesvirus reactivation from oral lesions to the skin of the pinna and outer ear canal (Hunt, 1907), and subsequently through neuroanatomical tracing (Semba et al., 1984). Several authors have since examined these auricular geniculate ganglion neurons regarding their developmental origin, expression of receptors for transmitter and neurotrophin receptors, and their passive electrical and firing properties (King and Bradley, 2000; Yamout et al., 2005; D’Autreaux et al., 2011). Although they are stated to be somatosensory, we are unaware of a functional demonstration of evoked responses in culture or by *in vivo* stimulation of the ear. We found that these auricular neurons responded to brushing, but not to gentle touch, deep pressure or changes of temperature (both heating and cooling). Thus, they appear similar to low-threshold mechanosensors of the skin. We previously obtained deep RNA sequence data from auricular neurons of the geniculate ganglion (Dvoryanchikov et al., 2017). Thus, we examined whether auricular neurons express the subtype-uniquely enriched genes (SUEGs) that were identified for DRG neurons (Zheng et al., 2019). The transcriptome of most of our sequenced auricular neurons was most similar, but not identical, to Aδ-low threshold mechano-receptors (Aδ-LTMRs). However, numerous SUEGs and genes ubiquitously expressed in Aδ-LTMRs were not expressed in auricular neurons (data not shown). Thus, it is possible that these auricular neurons form a somewhat distinct class of Aδ-LTMRs. This is consistent with their strong activation by brushing the ear, and lack of a visible Ca^2+^ signal for a static light touch with a flat probe. Based on our transcriptome data, a minority (≈20%) of the auricular neurons did not readily conform to previously defined mechanosensor types (Zheng et al., 2019). Consistent with the lack of thermosensitivity reported here, Trpv1, Trpv2, and Trpm8 are not expressed in geniculate auricular neurons (Dvoryanchikov et al., 2017), and they appear distinct from other mechanosensory or polymodal nociceptor neurons found in the dorsal root and trigeminal ganglia (Nguyen et al., 2017; Häring et al., 2018; Zheng et al., 2019).

## Synthesis

Reviewing Editor: Julie Bakker, University of Liege Decisions are customarily a result of the Reviewing Editor and the peer reviewers coming together and discussing their recommendations until a consensus is reached. When revisions are invited, a fact-based synthesis statement explaining their decision and outlining what is needed to prepare a revision will be listed below. The following reviewer(s) agreed to reveal their identity: Helene Bras.

Synthesis: the ms has been reviewed by 2 reviewers and both agree that the ms has been significantly improved. Just some minor revisions will be required.

Reviewer 1:

This is a much improved manuscript from the original submission. The authors attended appropriately to all of the original concerns of the reviewers. The paper now has the requisite rigor for this journal and it clearly tests a hypothesis about a cell type (auricular nerve cell) in a sensory ganglia (geniculate) containing multiple cell types that have been molecularly characterized.

Reviewer 2:

This study demonstrates that AAV-PHP.S, injected in the retro-orbital sinus of mice, is a powerfull tool to transduce multiple cranial sensory ganglia. From a functional point of view, the authors used a cre-dependent construct to perform in vivo Ca2+ imaging and characterize specifically chemico- or mechano-sensitive ganglion neurons. The results are convincing and well illustrated.

We have a few minor comments:

- References to the types of tast buds (fungiform vs circumvallate) should be quoted.

- Tast buds in one or two words (l547)

- Fig8 : a merge image of mCherry and GCaMP would be useful.
