# Supplementary figures and images for: Selectively Imaging Cranial Sensory Ganglion Neurons Using AAV-PHP.S
Source: eNeuro. 2022 Jun 3;9(3):ENEURO.0373-21.2022. doi: 10.1523/ENEURO.0373-21.2022 (PMC9186415; doi:10.1523/ENEURO.0373-21.2022)

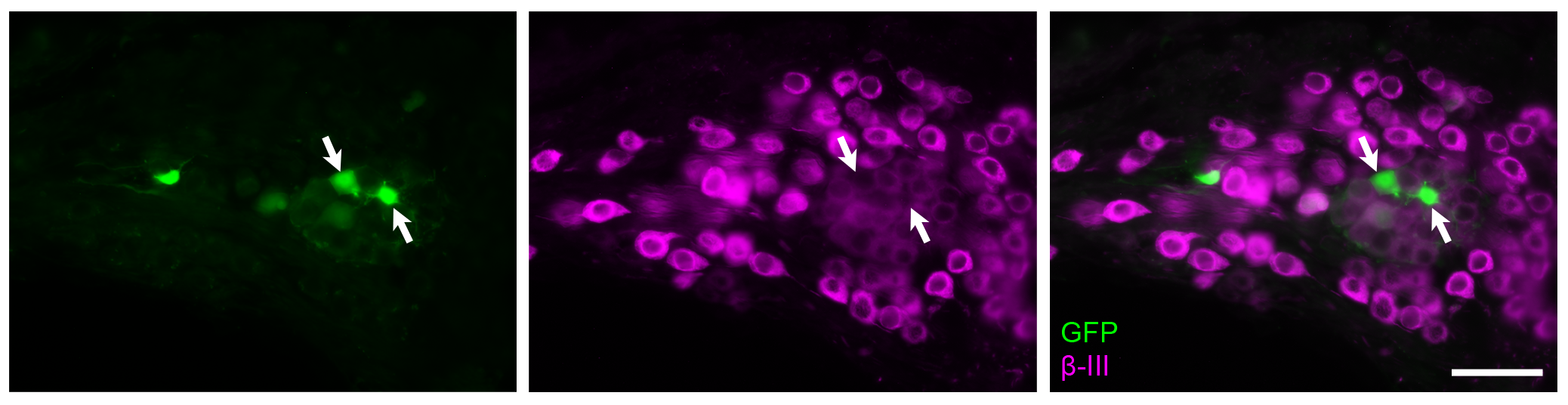

Supplement: Extended Data Figure 1-1 — AAV-PHP.S transduces a small number of cells in the spiral ganglion; 15-μm-thick cryosections of decalcified cochlea from mice infected with AAV-PHP.S::CAG-GFP were immunostained for βIII-tubulin (magenta). Those neurons which are robustly GFP-positive are also negative for βIII-tubulin (arrows). This suggests that AAV-PHP.S may be targeting the very few Type II pseudounipolar neurons present in the spiral ganglion, and avoiding the majority of βIII-tubulin-positive Type I bipolar neurons. Images are single plane. Scale bar: 20 μm Download Figure 1-1, TIF file. [file enu-eN-MNT-0373-21-s02.tif]

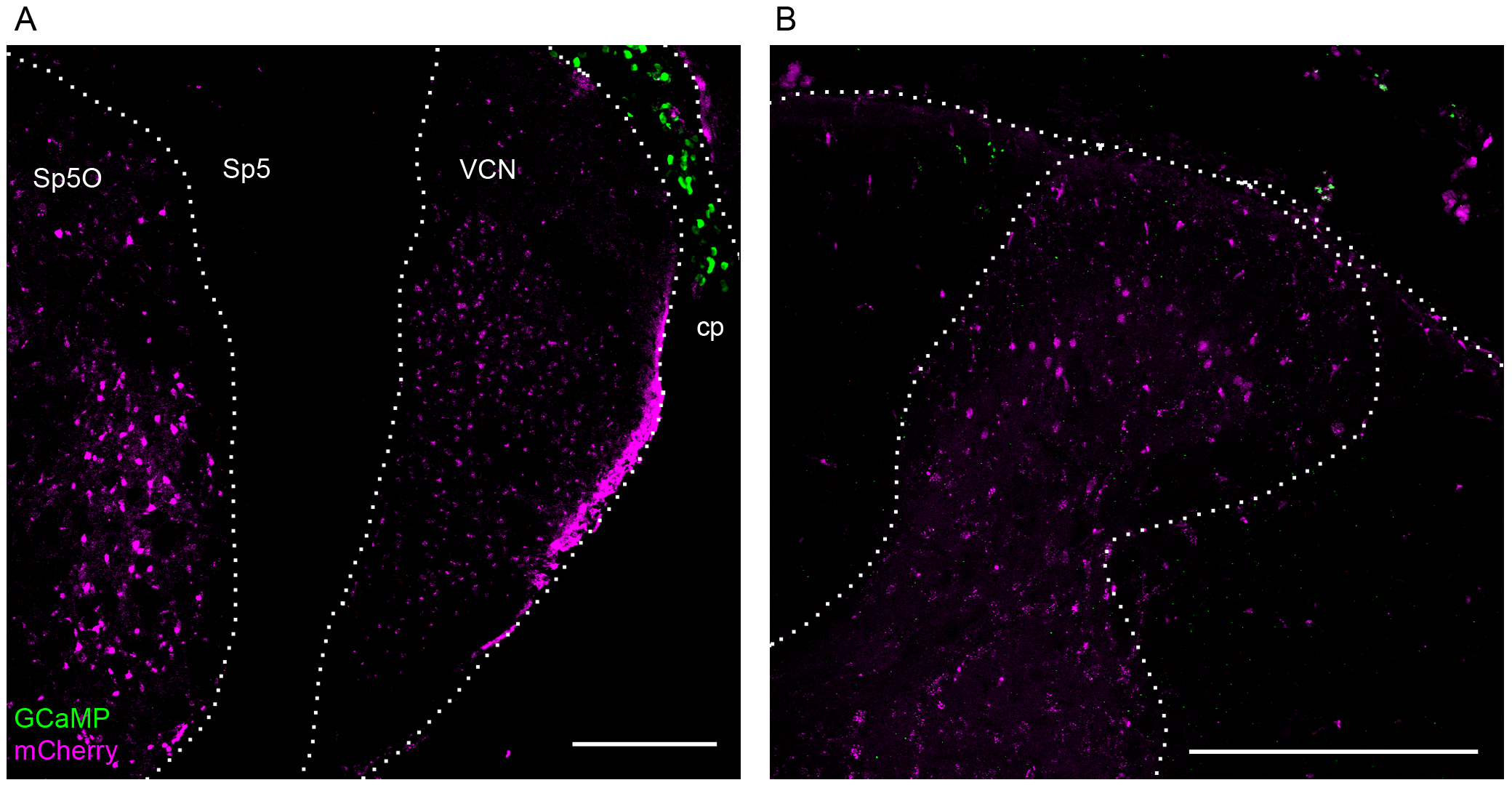

Supplement: Extended Data Figure 7-1 — Cre-dependent expression is limited to peripheral neurons. A Mafb-mCherry-Cre mouse was injected with AAV-PHP.S::flex-GCaMP6s and perfusion fixed 19 d later. A, Cryosection of hindbrain shows mCherry+ (i.e., Cre-expressing) central neurons of the spinal trigeminal nucleus, oral part (Sp5O) and the ventral cochlear nucleus (VCN). These remain untransduced and completely devoid of GFP. Some GCaMP6 is visible in the choroid plexus (cp) within the lateral recess of the 4th ventricle. B, Cryosection of thoracic spinal cord similarly shows many Cre-expressing neurons of the spinal gray and these remain untransduced. In both cases, the peripheral afferents lack Mafb and Cre expression, and thus also are unlabeled. Scale bars: 250 μm. Download Figure 7-1, TIF file. [file enu-eN-MNT-0373-21-s04.tif]
